# Cytoreductive surgery and hyperthermic intraperitoneal chemotherapy (HIPEC) for ovarian cancer in an Australian institution: lessons from 20 years’ experience

**DOI:** 10.1186/s12893-022-01786-7

**Published:** 2022-09-12

**Authors:** R. Farrell, W. S. Liauw, D. L. Morris

**Affiliations:** 1 Chris O’Brien Lifehouse Hospital, Camperdown, Sydney, NSW 2050 Australia; 2 The University of Sydney, Sydney, NSW 2006, Australia; 3 Department of Surgery, UNSW, Sydney, NSW 2033 Australia; 4 Cancer Care Centre, St George Hospital, Kogarah, NSW 2217 Australia; 5 Prince of Wales Private Hospital, Suite 30, Level 7, Randwick Sydney, 2031 Australia

**Keywords:** Ovarian cancer, Cytoreductive surgery, Hyperthermic intraperitoneal chemotherapy (HIPEC), Surgical complications

## Abstract

**Objectives:**

We report the 20-year experience of the largest Australian unit performing cytoreductive surgery (CRS) and hyperthermic intraperitoneal chemotherapy (HIPEC) for ovarian cancer and reflect on learning opportunities.

**Methods:**

A retrospective review of all cases of CRS for ovarian cancer at St George Peritonectomy Unit from Jan 1998 to Jan 2018 was performed. Prospectively collected data include age, stage, histology, disease extent (PCI), completeness of cytoreduction (CC score), HIPEC regime, 30-day surgical morbidity, disease recurrence, and death. Survival was computed using Kaplan–Meier method and analysed using log-rank tests and Cox-proportional hazards models.

**Results:**

Forty-one women with advanced ovarian cancer (11 primary stage III/IV, 30 recurrent) underwent CRS, 29 (71%) with HIPEC. Most (68%) had high-volume disease (PCI > 15). In 98%, CC0/CC1 (residual < 2.5 mm) was achieved. Fourteen (34%) had grade 3/4 complications, 1 patient (2%) died within 30 days and 2 patients (5%) died within 90 days. Progression-free and median overall survival was 30.0 and 67.0 months for primary cancer, and 6.7 and 18.1 months for recurrent cancer. Survival was associated with platinum-sensitivity, PCI ≤ 15, and CC score 0, but not HIPEC.

**Conclusion:**

This study reports outcomes for patients with advanced ovarian cancer patients treated in an Australian centre offering CRS and HIPEC. Whilst survival and morbidity outcomes were good for primary disease, they were poorer than predicted from the literature for cases of recurrent disease. The incorporation of evidence-based predictors of survival and multidisciplinary input are essential to achieve the best survival outcomes.

## Introduction

Ovarian cancer is the leading cause of gynaecological cancer death in Australia, with an age-standardised incidence of 10.6 per 100,000 women and 1047 women dying from this disease in 2017 [1]. Most cases are of epithelial high grade serous type, and arise from the ovary, fallopian tube, or peritoneum. In the majority (70%) at diagnosis, tumour has spread beyond the pelvis to the peritoneal surfaces of the abdomen. Despite efforts to improve outcomes, the 5-year relative survival in Australia for such cases is approximately 20–25% [2]. An effort to improve survival has led to the introduction of newer surgical and chemotherapeutic approaches, and more recently targeted treatments based on molecular and genetic characteristics. Internationally there has been an increase in the use of extended surgical procedures to achieve complete cytoreduction (extended CRS, or peritonectomy), combined with the use of hyperthermic intraperitoneal chemotherapy (HIPEC). This treatment modality has been used for other malignancies associated with peritoneal metastases including pseudomyxoma peritonei, colorectal cancer, and mesothelioma.

There is a large body of evidence supporting improved survival following maximal cytoreductive efforts for primary ovarian cancer [3, 4]. The use of nerve-sparing posterior exenterative procedures together with radical hysterectomy results in low pelvic recurrence rates with good functional outcomes [5]. The importance of incorporating surgical procedures to remove macroscopic disease in the upper abdomen, such as diaphragmatic stripping and total omentectomy, is well supported [6, 7]. In regards to recurrent ovarian cancer, for selected cases of first-relapse recurrent disease, survival outcomes might be improved if complete cytoreduction can be achieved [8–10].

In regard to HIPEC, there is evidence from one randomised controlled trial (RCT) [11], in addition to meta-analysis of prospective non-randomised trials [12], showing improved survival outcomes following HIPEC in women with stage 3 ovarian cancer. The Dutch-led RCT by van Driel [11] showed an 11.8 month improved overall survival in women who received HIPEC (Cisplatin 100 mg^2^ at 40 °C for 90 min) at the time of cytoreductive surgery following response to neoadjuvant chemotherapy. The results of HIPEC for recurrent ovarian cancer are less clear, with a recent small phase II RCT showing no benefit to HIPEC in patients with first recurrence of disease [13].

The use of extended CRS and HIPEC for ovarian cancer in Australia has been limited, with almost all such cases prior to 2018 being performed in one centre at St George Hospital in Sydney. This study is an analysis of the clinical and pathological characteristics, post-treatment morbidity, and survival outcomes of patients with primary and recurrent ovarian cancer treated with extended CRS and HIPEC at the St George Peritonectomy Unit over 20 years up until January 2018. The results will be discussed in context with current evidence and learning points that could help to shape future directions for this treatment modality.

## Materials and methods

A retrospective review of prospectively collected data from all consecutive cases of CRS, with or without HIPEC, for epithelial OC performed at St George Peritonectomy Unit between January 1998 and January 2018 was undertaken. Clinical and pathological data collected included age, ASA score, FIGO stage at diagnosis, histology, volume of disease measured by the peritoneal carcinoma index (PCI), and completeness of cytoreduction (CC score). The method of HIPEC used and type of chemotherapy given was recorded. Post-operative morbidity was recorded as type of complication and measured using the Clavien-Dindo classification of surgical complications [14].

Disease-free interval prior to CRS for recurrent disease was calculated from the most recent date of chemotherapy. “Platinum-sensitive” disease was defined as a disease-free interval of ≥ 6 months, whilst “Platinum-resistant” disease was defined as a disease-free interval of < 6 months. Progression-free survival was defined as the interval between date of CRS and the date of confirmation of recurrent or progressive disease by progressive rise in Ca 125 or new disease on imaging reviewed by the radiologist at the Peritonectomy MDT radiology meeting (on CT or PET CT scan). Survival was measured up until the date of death, or date of last contact.

Progression-free and median overall survival times were calculated using Kaplan–Meier method, and prognostic variables were analysed using log-rank tests and Cox proportional hazards model with SPSS v25. p values are derived from two-tailed tests. This study was conducted using an appropriate consent process and ethics process (HREC 18/067, SSA 18/G/092) and in accordance with the Helsinki Declaration (1983).

## Results

### Clinical, pathologic, and demographic characteristics

A total of 41 women underwent a peritonectomy procedure for advanced epithelial OC. Table 1 shows the clinical and pathological characteristics of the study cohort. The median age was 55 years (33–73 years). Median follow-up was 46.0 months. A second (re-do) CRS was performed in 4 women, 3 having a second surgery and one a third surgery. The majority of all cases (32/41, 78%) were high grade serous histology. In the 11 cases of primary OC, FIGO stage was IIIB (1), IIIC(8), and IV (2). Seven of the 11 primary cases received neoadjuvant platinum-based chemotherapy. Of the 11 primary cases, 6 cases received HIPEC with cisplatin, 5 did not receive HIPEC. Of the 30 cases of recurrent OC, disease was platinum-sensitive (PS) in 13 cases, and platinum-resistant (PR) in 17 cases. The recurrent group had received between one and 5 (median of 2) previous lines of chemotherapy before surgery. Twenty-three of the 30 recurrent cases received HIPEC;  19 with Cisplatin, 3 Mitomycin C, and one with Oxaliplatin. The median PCI of all cases was 22 (range 5–39), and PCI was greater than 15 in 28/41 (68%) of cases. A CC0 resection was achieved in 24/41 (59%), CC1 in 16/41 (39%), and CC2 in 1/41 (2%) of cases. HIPEC was given to a total of 29/41 (71%) of cases. If HIPEC was given the method used was the open, or colosseum technique, with median intraperitoneal temperature of 42 °C. Chemotherapy was Cisplatin in 25/41 (61%), Mitomycin in 3/41 (7%), and Oxaliplatin in 1 case. Four patients received early post-operative IP chemotherapy (EPIC) within the first 5 days (for median of 1 day). Adjuvant chemotherapy after discharge was received in 27/41 (66%) of patients, and in most cases was Carboplatin and Paclitaxel.

**Table 1:** Clinical and pathological characteristics of patients with advanced epithelial ovarian cancer undergoing peritonectomy with or without HIPEC

Variable	n = 41	Range
Age (median, years)	55.0	33–73
Follow-up (median, months)	46.0	
Length of stay (days)		
ICU	4.5	1–32
Hospital	29.3	7–82
Operating time (mean, h)	8.2	4–14

Variable	n = 41	n (%)
Presentation		
Primary	11	27
Recurrent	30	73
Tumour origin		
Ovarian	33	80
Fallopian tube	4	10
Peritoneal	4	10
Histology		
High grade serous	32	78
Endometrioid	0	0
Mucinous	3	7
Clear cell	1	2
Low grade serous	5	12
Peritoneal Cancer Index (PCI)		
≤ 15	13	32
> 15	28	68
Completeness of cytoreduction (CC score)		
0	24	59
1	16	39
2	1	2
3	0	0
HIPEC		
Yes	29	71
No	12	29
Complications (Clavien-Dindo)		
None	9	22
Grade 1/2	17	41
Grade 3/4	14	34
Death	1	2

### Morbidity and mortality

Grade 3 or 4 complications occurred in 14/41 (34%) of patients, as shown in Table 2. Almost all (13/14) cases with severe complications occurred in the recurrent cancer group. This consisted of 10/41 (24%) patients requiring re-operation, 9/41 (22%) with sepsis, and 6/41 (15%) with fistula. Two of the six fistulae were pancreatic, the remaining 4 cases were gastro-intestinal fistulae. The reason for re-operation was intra-peritoneal bleeding (4), small bowel obstruction (2), wound dehiscence (2), gastric perforation (1), pancreatic necrosis/abscess (1), wound dehiscence (2), and gastroscopy for dilatation (2). There were 4 (10%) with pulmonary embolus, and 2 patients suffered acute renal failure, with one of these patients requiring on-going intravenous electrolyte replacement following discharge. One woman died within 30 days and two women died within 90 days from surgery.

**Table 2:** Post-operative complications following peritonectomy and HIPEC in 41 patients with advanced epithelial ovarian cancer

Complication	n = 41	(N %)
Death in-hospital	1	(2)
Death in 90 days	2	(5)
Reoperation	10	(24)
Grade 3 complication (at worst)	5	(12)
Grade 4 complication (at worst)	9	(22)
Cardiac	1	(2)
Pulmonary		
Pleural effusion	12	(29)
Infection		
Sepsis	9	(22)
Pneumonia	3	(7)
UTI	3	(7)
Wound dehiscence	2	(5)
Intra-abdominal		
Haemorrhage	4	(10)
Fistula	6	(15)
Pancreatic leak	2	(5)
Perforated Viscus	1	(2)
Renal		
Acute renal failure	2	(5)
Thrombo-embolic		
Pulmonary embolism	4	(10)
DVT	1	(2)
Haematologic		
Blood Tx (average) units	7.3 units	

### Survival

**Fig. 1:**
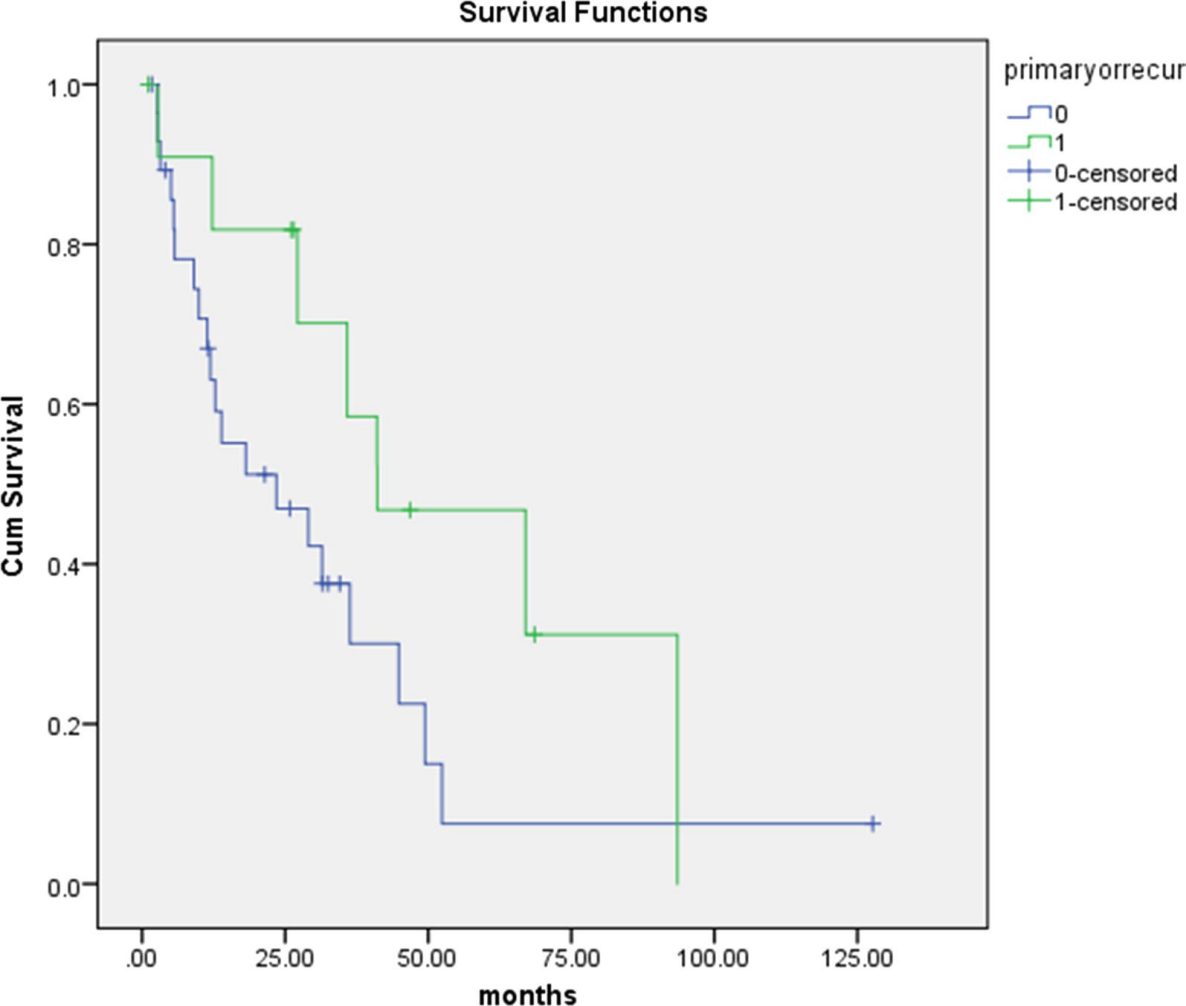
Kaplan–Meier curve for overall survival of 41 women with primary (n = 11) (green) and recurrent (n = 30) (blue) advanced epithelial ovarian/fallopian tube/peritoneal cancer following peritonectomy (with or without HIPEC)

Overall, 27/41 (66%) of women died during follow up, of which 6 were treated for primary disease and 21 for recurrent disease. There was one in-hospital post-operative death attributed to intra-abdominal sepsis in a patient who had CRS and HIPEC for recurrent disease. Two other patients died within 90 days post-operatively, both developing small bowel obstruction following CRS and HIPEC for recurrent ovarian cancer with a PCI of 26 and 39 respectively, both with CC1. The Kaplan Meier curve for overall survival of primary and recurrent disease is shown in Fig. 1.

The results for progression-free survival (PFS), median overall survival (OS), and 5-year median survival for both primary and recurrent ovarian cancer in the total group are shown in Table 3.

**Table 3:** Progression-free, median overall, and 5-year median survival for primary and recurrent advanced ovarian cancer following peritonectomy with or without HIPEC

Survival	Primary OC n = 11	Recurrent OC n = 30	Platinum Sens* n = 13	Platinum Res** n = 17
Progression-free (months)	30.0	6.7	30.8	6.5
Median overall (months)	67.0	18.1	31.5	12.8
5-year (%)	27.0	3.0		

^*^Platinum Sens = platinum sensitive recurrent disease

^**^Platinum Res = platinum resistant recurrent disease

On log-rank analysis, survival of the total group was significantly associated with PCI and CC score. Median overall survival was higher in the PCI ≤ 15 versus > 15 group (respectively 49.5 months versus 12.8 months, p = 0.015), and in the CC0 compared to the CC1 or CC2 group (41.1 months versus 13.9 versus 3.2 months, p = 0.006).

For the 30 cases of recurrent disease, survival was significantly better in the platinum- sensitive compared to the platinum-resistant group (31.5 versus 12.8 months, p = 0.04). (Table 3). On multivariate analysis, the prognostic variables associated with significantly longer survival were primary rather than recurrent disease, a lower PCI, and CC score of 0. The use of HIPEC did not significantly improve survival in the total cohort on univariate or multivariate analysis (Table 4).

**Table 4:** Survival differences for prognostic variables using log-rank analysis

Variable	Value	Median overall survival (months)	95% CI	p value
PCI	≤ 15	49.5	31.5–67.4	
	> 15	12.8	3.3–22.3	***0.015***
CC score	0	41.4	24.5–57.6	
	1	13.9	11.0–16.8	
	2	3.2		***0.006***
Recurrent disease	n = 30	18.1		
Platinum sensitive	n = 13	31.5	23.1–39.8	
Platinum resistant	n = 17	12.8	9.3–16.3	***0.04***

All *p* values < 0.05 are shown in bold

*PCI* peritoneal cancer Index, *CC score* completeness of cytoreduction score

## Discussion

The St George Peritonectomy Unit in Sydney has the largest national experience of cytoreductive surgery and HIPEC for ovarian cancer and up until January 2018 had performed most cases within Australia. The unit has extensive experience in peritonectomy surgery, having performed over 1200 surgeries for peritoneal carcinomatosis up until that time. The surgical team is led by a surgical oncologist/hepatobiliary surgeon and the surgeons are surgical oncologists/general surgeon trainees. The medical oncologists have had considerable expertise in treating mostly GIT-related cancers. The unit operates outside of gynaecological cancer centres located in Australia and receives around 1.5% of all ovarian cancer cases in the state of NSW[15].

The ovarian cancer cohort cared for in this unit had quite advanced disease, shown by the fact that 68% of cases had a PCI above 15. Despite this, our study showed that in 98% of cases, residual disease was less than 2.5 mm. These results reflect the radicality of surgery used by an experienced surgical oncology team and compares favourably to a reported “optimal debulking” rate of 60% by Australian gynaecological oncologists in a recent study performed by this author [16]. It is well recognised that one of the strongest predictors of survival following surgery for ovarian cancer is the ability to resect all macroscopic disease [4]. To achieve this goal, surgery for ovarian cancer should be conducted in a unit with the required collaborative expertise to proficiently remove disease from both the pelvis and abdomen.

The results for PFS and median OS for primary disease in this study are comparable to published results for long-term follow-up of primary CRS with post-operative IP chemotherapy [17]. Using data obtained from 876 patients randomised to adjuvant IP chemotherapy or IV chemotherapy following CRS for primary ovarian cancer, at a median follow-up of 10.7 years, Tewari et al. [17] found a median OS of 61.8 months for IP chemotherapy compared to 51.4 months for those given IV chemotherapy. This compares to a median OS of 67.0 months in our study. This also compares favourably with a median OS of 39.0 months in a population-based study of 1452 unselected cases of stage 3/4 epithelial ovarian/tubal/peritoneal cancer treated between 2002 and 2013 recently reported by an Australian gynaecological oncology centre (Queensland Centre for Gynaecological Cancer) [18]. Whether the superior survival shown in the St George cohort compared to the Australian gynaecological cancer centre is due to a higher rate of complete cytoreduction, the application of HIPEC, or is a result of case selection or other bias is not possible to determine.

As expected, survival was significantly lower in patients with recurrent ovarian cancer than in those with primary disease. In the recurrent group (n = 30), median PFS was 6.7 months and median OS was 18.1 months. This compares unfavourably to a median PFS of 19.6 months and median OS of 53.7 months in the surgery arm of the AGO DESKTOP III/ENGOT ov20 study [8]. The DESKTOP III study is one of three recently published randomized trials  (GOG 213 (2019) [9], DESKTOP III (2020) [8], and SOC 1 (2021) [10]) comparing secondary CRS to chemotherapy alone in women with recurrent platinum-sensitive OC. Results from these studies show that strict selection criteria is essential if any survival benefit is to be achieved from secondary CRS compared to chemotherapy alone. The factors shown to be predictive of a survival benefit in the DESKTOP III and SOC 1 trials were platinum-sensitivity (≥ 6 months platinum-free interval), absence of significant co-morbidities (ECOG 0), absence of large ascites (< 500 mls), and complete resection of disease. In comparison, patients in the St George group had received between 2 and 5 lines of chemotherapy before surgery, and the majority (17/30, 57%) had platinum-resistant (PR) disease.

The significantly poorer outcomes for platinum-resistant disease (median OS 12.8 months) supports the exclusion of these cases from secondary CRS, at least outside of a well-designed clinical trial. There may be a subgroup of women with PR disease that could benefit from secondary CRS such as those with isolated/recurrent disease, low grade serous carcinoma, or those requiring palliative procedures (e.g.: to relieve bowel obstruction) [19]. However, international efforts are required to conduct multicentre prospective clinical trials to clarify whether surgery can offer any benefit to these patients over available second/third line chemotherapy or targeted treatments (including PARP inhibitors or bevacizumab).

In our study, the use of HIPEC was not associated with improved survival. Our study was underpowered to measure this outcome. The best evidence currently available to support HIPEC for the treatment of primary ovarian cancer is the RCT of van Driel et al. [11], which included 245 women with at least stable disease following 3 cycles of NAC randomised to either CRS, or CRS plus HIPEC. This study showed an 11.8 month improved median OS when HIPEC was given (45.7 months versus 33.9 months, HR = 0.66, 95% CI 0.50–0.87, p = 0.003). Moreover, rates of grade 3/4 complications in the van Driel study were similar between the HIPEC and no HIPEC arms (27% vs 25%, p = 0.76, respectively). Despite a higher rate of stoma in the HIPEC arm (72 vs 43%, p = 0.04), health related QOL did not differ between the groups [20]. The more recent but smaller RCT by Lim [21] showed improved survival following HIPEC only in a sub-group of women who received interval CRS, but not in the primary CRS group. These results are encouraging, and there are currently over 8 ongoing RCTs of HIPEC for primary ovarian cancer. This includes an Australian-led RCT comparing HIPEC to normothermic intraperitoneal chemotherapy (NIPEC) for primary advanced OC (HyNOVA) [22].

There is limited evidence, however, that HIPEC can improve survival in women with recurrent OC. There are 2 RCTs that have compared HIPEC to no HIPEC at the time of secondary CRS for recurrent ovarian cancer. The first RCT by Spiliotis [23] showed improved survival in patients with recurrent OC given HIPEC, but this study has since been discounted due to significant faults in methodology and analysis [24]. A recent RCT by Zivanovic et al. [13] included 98 women with first recurrence of high grade epithelial ovarian cancer (97% had HGSOC). Patients were randomised intra-operatively to receive HIPEC (carboplatin), or no HIPEC, if the surgeon confirmed $\le$ 0.5 cm residual disease. Complete resection was achieved in 88% of patients, and both groups received standardised post-operative chemotherapy. Results of this study showed no significant difference in disease recurrence at 24 months, or median OS (52.5 months versus 59.7 months, HR = 1.39, 95% CI 0.73–2.67, p = 0.31). Although the study has limited power due to a small sample size, the findings do not support the use of HIPEC at the time of secondary CRS for platinum-sensitive recurrent ovarian cancer.

The potential for increased rates of post-operative morbidity and mortality with an aggressive approach to surgery is important to consider. In our study, one-third (34%) of patients had grade 3/4 complications, with a need for re-operation in almost one quarter (24%), and a fistula rate of 15%. All but one grade 3/4 complication occurred in the recurrent cancer group, and all 3 women who died within 90 days of surgery were in the recurrent group.

Morbidity following surgery for advanced ovarian cancer depends on the complexity of surgery, the experience of the surgeon/unit, and individual patient factors (including age, co-morbidities, and albumin level) [25]. The performance of multiple visceral resections is one of the strongest risk factors for severe morbidity. In a study of 2870 women undergoing ovarian cancer surgery from the NSQIP database between 2005–2012, Patankar et al. [26] found that the rate of overall complications increased from 7.3% in those that did not require any extended procedures, to 12.9% after one procedure, to 30% for ≥ 3 extended procedures. Most of the patients in our study had multiple (≥ 3) extended procedures, particularly in the recurrent cancer group, so a rate of grade 3/4 complications of 34% is consistent with this finding. In particular, there were 2 cases of pancreatic leak with fistula in our study, both in patients with recurrent high-volume (PCI 39 and 27) disease requiring splenectomy and treated with HIPEC. In a previous retrospective study of 260 patients with advanced or recurrent ovarian cancer who had a splenectomy performed during CRS, HIPEC was identified as a significant risk factor in those who underwent splenectomy without concomitant pancreatic surgery [27].

There is evidence from four RCTs [28–31] that giving neoadjuvant chemotherapy (NAC) to selected patients with advanced ovarian cancer can decrease surgical morbidity without adversely affecting survival. The SCORPION trial [31], an RCT comparing up-front surgery to NAC followed by interval CRS in 171 women with high tumour load, showed that the use of NAC decreased the need for radical resections, increased the rate of complete resection from 47.6 to 67%, and significantly decreased the rate of severe post-surgical morbidity at 30 days from 46.4 to 9%. In our cohort, 7 of 11 patients with primary disease received NAC.

The use of NAC in patients with high tumour load can reduce tumour volume and reduce surgical morbidity. It can also allow optimisation of prehabilitation for patients who may initially be unfit for surgery and assist in the identification of those patients who are truly unlikely to benefit from CRS and HIPEC. This would include those patients with serious co-morbidities that do not improve with medical treatment, or those who progress whilst on chemotherapy. The TRUST (Trial of Radical Up-front Surgical Therapy in advanced ovarian cancer) study [32], an international RCT comparing primary CRS with interval CRS for stage IIIB-IVB ovarian cancer, should help to further define the role of extended surgical procedures with and without NAC in the management of advanced ovarian cancer. The estimated completion date for this trial is April 2023.

The significant independent predictors of survival in our study were the volume of disease (PCI), and the ability to achieve zero macroscopic disease (CC0), which are findings similar to most previous studies of ovarian cancer surgery either with or without HIPEC [4, 12]. The ability to predict both PCI and CC0 pre-operatively is therefore vital and relies on an experienced team. At the St George peritonectomy unit, weekly radiology meetings attended by radiologists with a high level of experience in detecting peritoneal disease on CT, MRI and PET CT scans are used as the triage tool to predict PCI and CC0. Although diagnostic laparoscopy (DL) was not routinely used, there is now a body of evidence to show that the use of DL in patients pre-operatively to assess tumour volume and distribution can also help triage patients to the most appropriate treatment pathway [33]. DL has been shown to be superior to CT imaging in predicting tumour volume in the pelvis and small bowel serosal disease. Rutten et al. [34] showed that DL used to triage patients to NAC rather than up front surgery reduced ‘futile’ laparotomies from 39 to 10%. DL also provides the opportunity to retrieve sufficient fresh tissue for somatic mutation testing prior to neoadjuvant chemotherapy, which is important to guide adjuvant targeted treatment options for ovarian cancer.

Treatments that target the molecular and genetic characteristics of ovarian cancer and the potential they have to significantly impact survival are important to consider when planning the optimal treatment pathway for each patient. Multiple studies in both primary and recurrent ovarian cancer have shown significant survival benefits from the use of Poly (ADP-ribose) polymerase inhibitors (PARP) inhibitors, particularly in those patients with a germline or somatic mutation in BRCA or similar genes associated with a defect in DNA repair known as homologous recombination deficiency (HRD) [35–41]. A recent update of the SOLO 1 trial [35], which randomised women with BRCA-mutated primary ovarian cancer to maintenance olaparib versus placebo following response to primary chemotherapy, showed that 48.3% of the group receiving olaparib were disease-free at 5 years, compared to 20.5% of the placebo arm. Given such enormous improvements in outcomes with PARP inhibitors means that future studies of CRS and HIPEC for ovarian cancer must incorporate molecular and genetic characteristics of disease, and the use of targeted treatments, into trial protocols and analysis. In GOG 213 [9], for example, bevacizumab (an angiogenesis inhibitor) was given to 84% of the study participants. This has been proposed as one reason why there was no benefit seen in the secondary CRS group compared to the chemotherapy-only group.

We recognise that this study is a single-centre study with a relatively small number of patients, with heterogenous disease and unknown genetic and molecular tumour characteristics. However, there are important learning points that can be made from this study when it is considered in the context of results from contemporary randomised clinical trials in ovarian cancer. The main learning points are;

1. Collaboration between expert surgical groups and optimisation of surgical training and experience can achieve high rates of complete cytoreduction, one of the most important factors influencing survival in ovarian cancer.
2. Careful selection of patients for up-front primary CRS (or interval CRS, or no surgery), and for secondary CRS (versus chemotherapy alone, and/or targeted treatments), is essential. Case selection will require an expert knowledge of the disease, which exists within a gynaecological cancer-specific multidisciplinary team framework.
3. A knowledge of the molecular and genetic characteristics of ovarian cancer, and factoring this knowledge into decision-making around surgery and adjuvant treatments, is essential to achieve the best survival outcomes.
4. Quality of life and survivorship outcomes, including fertility preservation, symptom control, and social and psychological needs of patients with ovarian cancer, require a specialised holistic approach to peri-operative and ongoing care.
5. Quality clinical trials, particularly RCTs, are required to further elucidate the benefit or otherwise of extended surgery and HIPEC for advanced ovarian cancer.

## Conclusion

This study presents a 20 year experience in the use of extended CRS and HIPEC in the treatment of women with advanced ovarian cancer in Australia. The importance of considering evidence-based predictors of survival and multidisciplinary collaboration are paramount to achieving the best outcomes.

## Data Availability

All data included in this study is available on request to the corresponding author.
